# Genomic characterization of extended-spectrum β-lactamase-producing *Escherichia coli* spread among chickens and healthy residents in Lombok, Indonesia

**DOI:** 10.1128/aem.02364-24

**Published:** 2025-04-11

**Authors:** Eustachius Hagni Wardoyo, Yo Sugawara, Satoshi Nakano, Hui Zuo, Shaheem Elahi, Chika Arai, Kohei Kondo, Kuntaman Kuntaman, Motoyuki Sugai

**Affiliations:** 1 Antimicrobial Resistance Research Center, National Institute of Infectious Diseases 13511, Tokyo, Japan; 2 Microbiology Department, Faculty of Medicine and Health Sciences, University of Mataram, Lombok, Indonesia; 3 Department of Microbiology, Faculty of Medicine, University of Wijaya Kusuma Surabaya 508024 , Surabaya, Indonesia; Centers for Disease Control and Prevention, Atlanta, Georgia, USA

**Keywords:** extended-spectrum β-lactamase, *Escherichia coli*, whole-genome sequencing, chicken, *bla*_CTX-M-55_, IncHI1 plasmid, indigenous chicken farming, Lombok, Indonesia

## Abstract

Extended-spectrum β-lactamase-producing *Escherichia coli* (ESBL-Ec) poses a substantial public health challenge, particularly in developing countries where antimicrobial resistance is a growing concern. Although extensive research has documented the prevalence and impact of ESBL-Ec in clinical settings and farm animals, the intricate relationship between poultry farming practices and human health remains unclear. To elucidate the genomic relationships between isolates from chickens and humans, we conducted a whole-genome sequencing analysis of 200 *E. coli* isolates derived from chickens, chicken farmers, and non-farmer volunteers in Lombok, Indonesia. The isolates exhibited considerable diversity, with 78 distinct sequence types (STs) identified by multilocus sequence typing. Of these, 35% (*n* = 70) and 6% (*n* = 12) were positive for ESBL and AmpC-type β-lactamase genes, respectively, with *bla*_CTX-M-55_ being the most prevalent (*n* = 41). The most prevalent STs among those carrying ESBL or AmpC genes were ST48, ST1485, ST1727, and ST2690. Phylogenetic analysis of publicly available data indicated that ST1485, ST1727, and ST2690 strains had an indigenous spread in this region, whereas the ST48 isolates originated from different sources. The *bla*_CTX-M-55_ gene was identified in 22 different STs, predominantly carried by an IncHI1A-type plasmid, suggesting horizontal transmission of the plasmid over the clonal barrier. Long-read sequencing analysis of representative isolates revealed the presence of additional resistance genes against different classes of antibiotics, suggesting that the acquisition of the IncHI1A-type plasmid contributes to multidrug resistance in recipient clones. Our analysis provides unique insights into the dissemination of multidrug-resistant ESBL-Ec strains in Lombok, where humans and chickens are in close proximity.

**IMPORTANCE:**

We performed a genomic comparison of ESBL-producing *E. coli* (ESBL-Ec) isolated from chickens, chicken farmers, and non-farmers on Lombok Island, where indigenous chicken farming, which involves close proximity of humans and chickens, is a major industry. The detection of the same ESBL-Ec clones in both chickens and farmers indicated the potential of a zoonotic transmission pathway for antibiotic-resistant bacteria. Moreover, the presence of a common plasmid carrying an ESBL gene along with other antimicrobial resistance genes in various *E. coli* clonal groups highlights the dissemination of resistance determinants within both poultry and human populations. This cross-species amplification of antimicrobial resistance poses a substantial risk to public health, as it can lead to the proliferation of multidrug-resistant bacterial infections, complicating treatment options and increasing the burden on healthcare systems. Addressing these issues is crucial for implementing effective antimicrobial stewardship and improving biosecurity practices in poultry farming.

## INTRODUCTION

The dissemination of antimicrobial-resistant (AMR) organisms is a substantial global public health concern, particularly due to the potential for these organisms to develop resistance to multiple antimicrobial agents. Extended spectrum β-lactamase (ESBL)-producing *Escherichia coli* (ESBL-Ec), which is resistant to penicillins and third-generation cephalosporins, is among the most prevalent AMR bacteria and constitutes the major global burden of diseases caused by the AMR bacteria (1). The recent revision of the WHO Bacterial Priority Pathogen List has upgraded third-generation cephalosporin-resistant *Enterobacterales* to the critical pathogen category owing to its considerable burden in low-income countries (2). ESBL-Ec is disseminated throughout the human population, which is a cause for concern given that ESBL-Ec colonization frequently precedes the onset of invasive infections (3). ESBL genes encompass a range of β-lactamases, with CTX-M family enzymes currently representing the most prevalent worldwide (4). These genes are frequently carried by transmissible plasmids, facilitating their dissemination among diverse *E. coli* genotypes or species (4).

The dissemination of AMR bacteria in food is another serious concern. The level of antimicrobial consumption in livestock has surpassed that in humans, which can lead to the emergence of AMR bacteria in the veterinary sector in addition to clinical settings, and, in turn, poses a risk of further spread of AMR bacteria in communities and clinical settings (5). Poultry is the most common meat source globally, with production occurring in a multitude of countries (6). The presence of ESBL-Ec in poultry has been extensively studied (7); however, the complex relationship between poultry farming practices and human health remains underexplored, particularly in developing countries. The recent application of whole-genome sequencing in AMR surveillance has enabled the analysis of AMR transmission between humans and poultry. However, the results of such surveillance studies differ among reports. Several reports have indicated that genetically closely related ESBL-Ec strains are shared between humans and poultry (8–10), whereas other studies have reached the opposite conclusion (11–13). These conflicting observations suggest that the pattern of spread of ESBL-Ec differs depending on the environment in which poultry are reared, namely intensive, non-intensive, or industrially well-regulated, as well as the manner in which poultry meat is processed. Indigenous chicken farming represents a substantial sector in the Indonesian island of Lombok. Most chicken farmers receive day-old chickens from employers and raise them in semi-open chicken coops or pens located in the same yard as the farmer’s house (14). In 2021, the total chicken population on the island was approximately 10,000,000 in Lombok (15). The chicken slaughterhouse was owned by an employer, which resulted in farming, slaughtering, and shipping on the same farm.

In this study, we used whole-genome sequencing to analyze ESBL-Ec colonizing chickens, chicken farmers, and non-farmers on the Indonesian island of Lombok. This approach allowed us to elucidate the patterns of the spread of this AMR organism in situations where humans and chickens were in close proximity.

## RESULTS

A total of 200 *E. coli* isolates were obtained from cloacal swabs from healthy chickens (*n* = 121, 60.5%) and rectal swabs from healthy farmers (*n* = 35, 17.5%) and non-farmers (*n* = 44, 22.0%) residing outside the chicken farm. Non-farmer samples were included to evaluate the role of direct contact between chickens and humans in the spread of ESBL-Ec.

**Fig 1:**
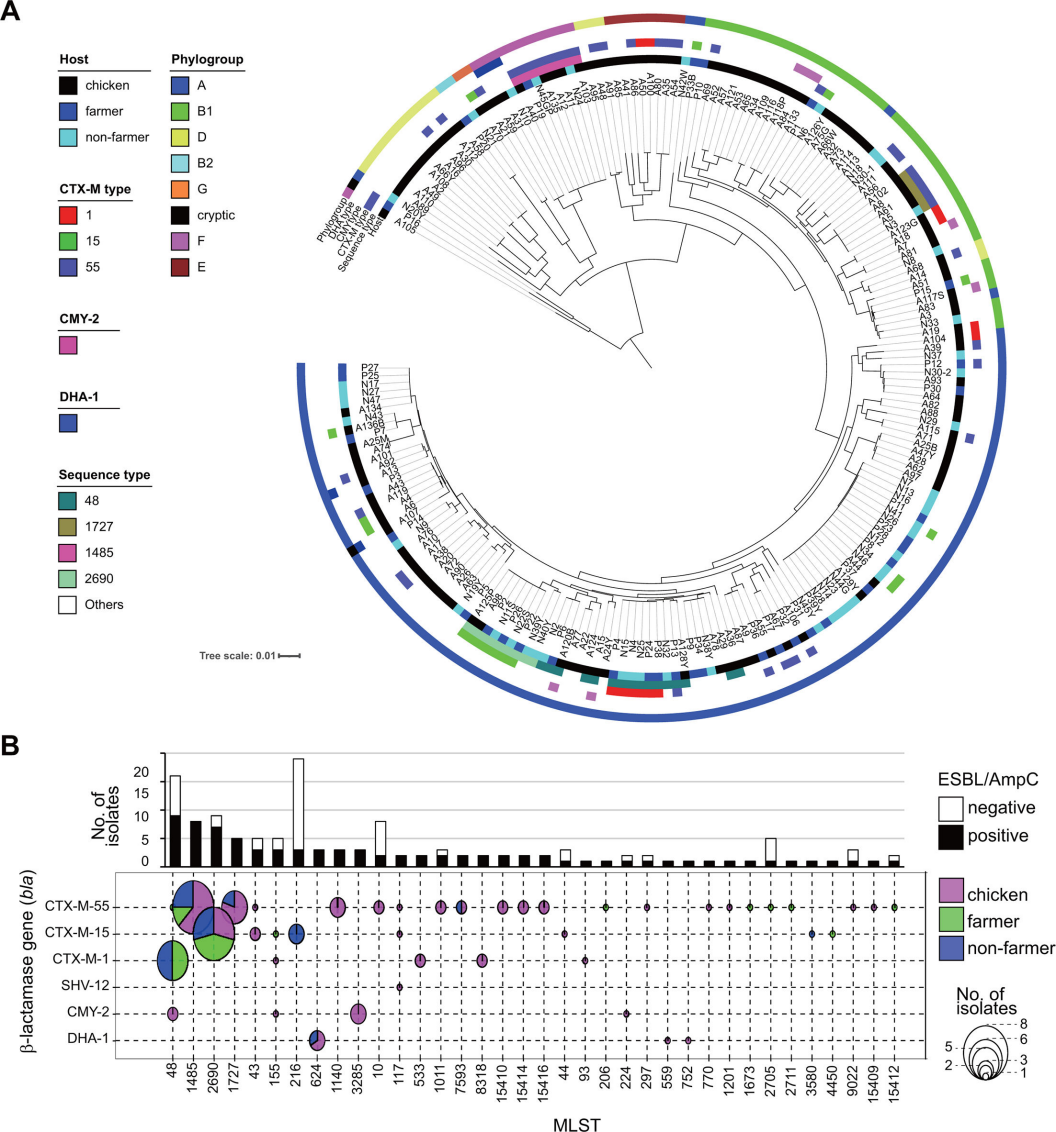
Phylogeny and ESBL/AmpC carriage of the Lombok *E. coli* isolates. (**A**) Phylogenetic tree of 200 isolates from chicken, farmer, and non-farmer analyzed in this study. (**B**) The upper graph shows the number of isolates with (black) or without (white) ESBL/AmpC carriage per ST. In the lower graph, vertical lines correspond to STs; horizontal lines correspond to ESBL or AmpC genes. The size of the circles reflects the number of strains, and different colors show different origins. ESBL, extended-spectrum beta-lactamase; ST, sequence type.

Whole-genome sequencing of the isolates revealed a high degree of clonal diversity, with 78 distinct sequence types (STs) identified by multilocus sequence typing (MLST), including nine newly assigned types (Fig. 1A). Phylogenetic groups A and B1, the predominant commensal groups in humans and animals (16), were the most prevalent among these isolates. A total of 13 and 12 STs were commonly identified in chickens and farmers, and chickens and non-farmers, respectively (Fig. S1). Of these, nine were common across all three origins. The ESBL gene was found in 70 isolates (35%), with positivity rates of 34.7% (42/121) in chickens, 42.9% (15/35) in farmers, and 29.5% (13/44) in non-farmers. Four ESBL types were identified in descending order of number: *bla*_CTX-M-55_ (*n* = 41), *bla*_CTX-M-15_ (*n* = 17), *bla*_CTX-M-1_ (*n* = 12), and *bla*_SHV-12_ (*n* = 1). One isolate co-harbored *bla*_CTX-M-55_ and *bla*_SHV-12_. AmpC β-lactamase genes were also identified in 12 isolates (6%). Two AmpC genes, *bla*_CMY-2_ and *bla*_DHA-1_, were identified in seven and five isolates, respectively. These genes were found exclusively in chicken isolates, with the exception of one farmer isolate carrying *bla*_DHA-1_. The most prevalent STs among those carrying ESBL or AmpC genes in this study were ST48, ST2690, ST1485, and ST1727 (Fig. 1B). Notably, *bla*_CTX-M-55_ was the most prevalent gene in ST1485, although it was also detected in 22 other distinct STs.

Antimicrobial susceptibility testing of the isolates positive for ESBL/AmpC genes showed that the antimicrobial susceptibility pattern was similar between chicken and human (farmer and non-farmer) isolates (Fig. S2A and B). However, the susceptibility rates of chicken isolates to gentamicin, tobramycin, levofloxacin, and minocycline were 26%, 29%, 21%, and 26% lower than those of human isolates, respectively. Thirty-five of the 42 gentamicin- and tobramycin-resistant isolates (83.3%) co-harbored *bla*_CTX-M-55_ which was detected in 56.6% and 37.9% of chicken and human isolates, respectively, carrying the ESBL/AmpC gene. The susceptibility rates to gentamicin and tobramycin in isolates with *bla*_CTX-M-55_ were clearly lower than those in isolates with other ESBL/AmpC genes (Fig. S2D). It was likely that aminoglycoside resistance genes co-harbored with *bla*_CTX-M-55_ dominantly mediate non-susceptibility. Details of the plasmids carrying *bla*_CTX-M-55_ are described below. The difference in susceptibility to levofloxacin and minocycline appeared to reflect the difference in the rate of mutations in the quinolone resistance-determining region (QRDR) and *tet(B*) carriage, respectively, between the chicken and human isolates (Fig. S2C). These resistance determinants were not associated with specific genotypes but were found in 21 and 14 different STs for QRDR and *tet(B*), respectively.

**Fig 2:**
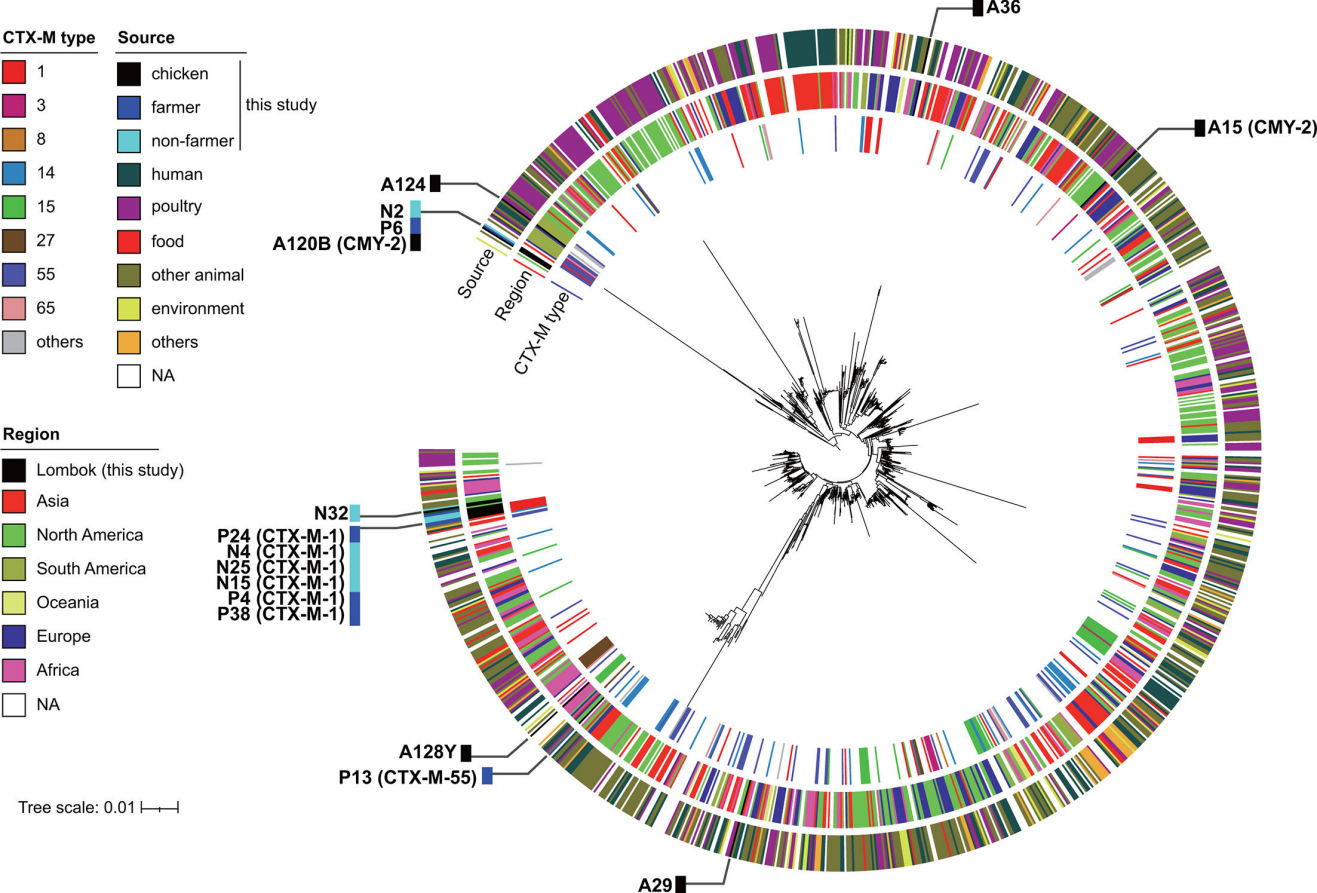
Phylogenetic tree of *E. coli* ST48 isolates obtained in this study (*n* = 16) and those publicly available from the database (*n* = 1,188). The former isolates were labeled outside the tree, and β-lactamase genes (*bla*) carried by these isolates are shown in parentheses. NA, not assigned.

**Fig 3:**
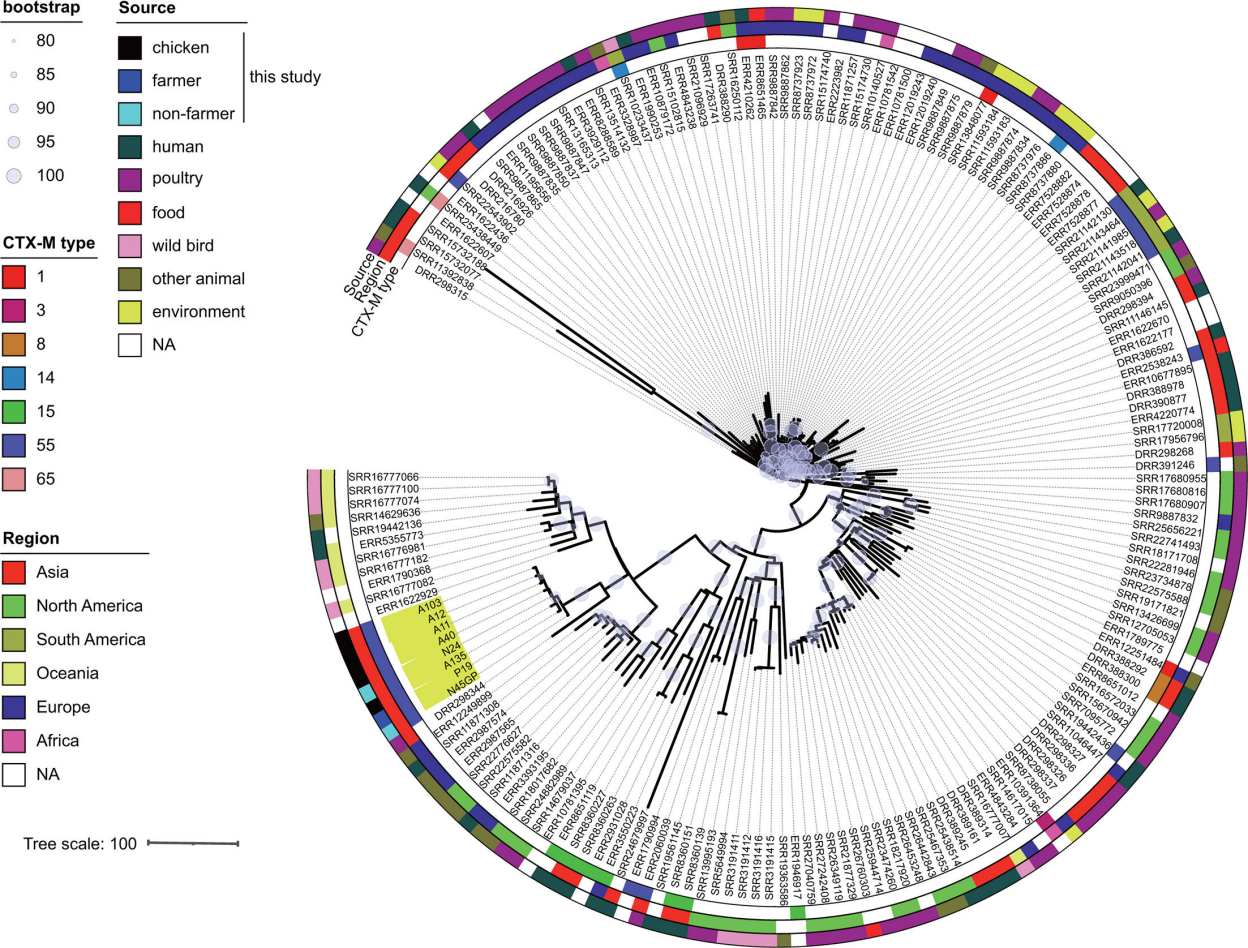
Phylogenetic tree of *E. coli* ST1485 isolates obtained in this study (*n* = 8, colored yellow) and those publicly available from the database (*n* = 171). NA, not assigned.

**Fig 4:**
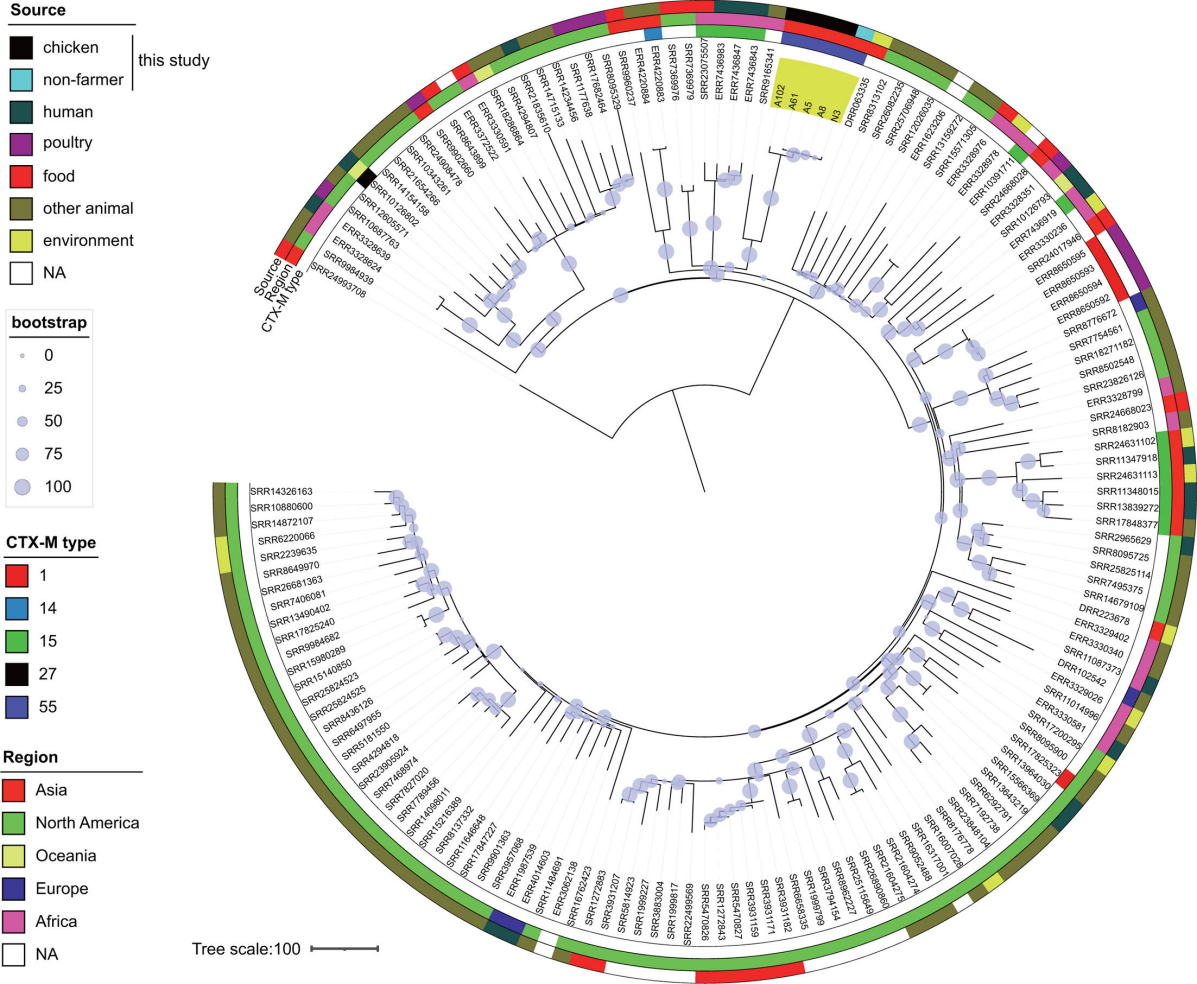
Phylogenetic tree of *E. coli* ST1727 isolates obtained in this study (*n* = 5, colored yellow) and those publicly available from the database (*n* = 150). NA, not assigned.

**Fig 5:**
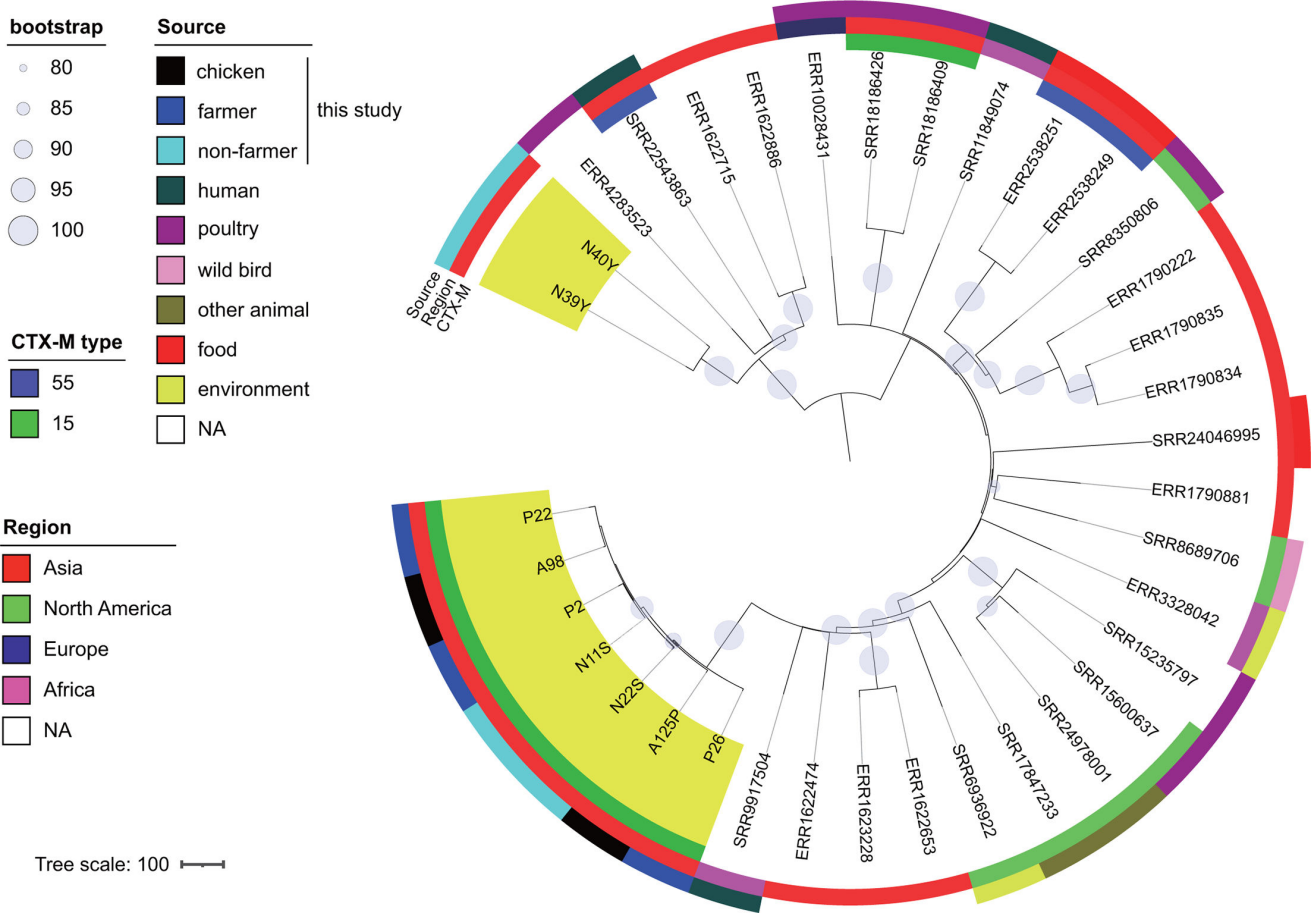
Phylogenetic tree of *E. coli* ST2690 isolates obtained in this study (*n* = 9, colored yellow) and those publicly available from the database (*n* = 27). NA, not assigned.

As described above, ST48, ST2690, ST1485, and ST1727 were the most prevalent STs among isolates carrying ESBL or AmpC genes and were detected in chicken and human sources. To gain insight into the evolutionary relationships of these isolates from a global perspective, we constructed a phylogenetic tree of the four major STs using the genome sequencing data of the isolates from this study and those from publicly available sources. Notably, a significant proportion of the isolates assigned to these four STs were reported from a range of geographical locations, with a substantial number originating from poultry and other animals (Fig. S3). The Lombok ST48 isolates (highlighted outside the circle in Fig. 2) were found in disparate branches of a phylogenetic tree, indicating that they diverged significantly from one another. Farmer and non-farmer isolates with *bla*_CTX-M-1_ clustered on the tree, suggesting that it was spread among Lombok residents. The *bla*_CMY-2_ gene was identified in two phylogenetically distinct chicken isolates. In contrast to ST48, Lombok isolates of other STs clustered together on the phylogenetic tree, indicating the regional dissemination of each strain (Fig. 3 to 5). Isolates of the Lombok ST1485 and ST1727 lineages carrying *bla*_CTX-M-55_ clustered together in each phylogenetic tree, regardless of their origin (Fig. 3 and 4). In the ST1485 cluster, the average single nucleotide polymorphism (SNP) differences between chicken isolates, human isolates, and between chicken and human isolates were 39.8, 31.2, and 26, respectively. Thus, chicken and human isolates were indistinguishable based on SNP differences (Fig. S4A). Similarly, in the ST1727 cluster, the average SNP differences among chicken isolates were 50.1, whereas the differences between chicken and human isolates were 29.8 (Fig. S4B). For ST2690, isolates with and without *bla*_CTX-M-15_ clustered separately. The first cluster contained human and chicken isolates (Fig. 5), with average SNP differences of 16 among chicken isolates, 11.2 among human isolates, and 12.3 between chicken and human isolates (Fig. S4C).

**Fig 6:**
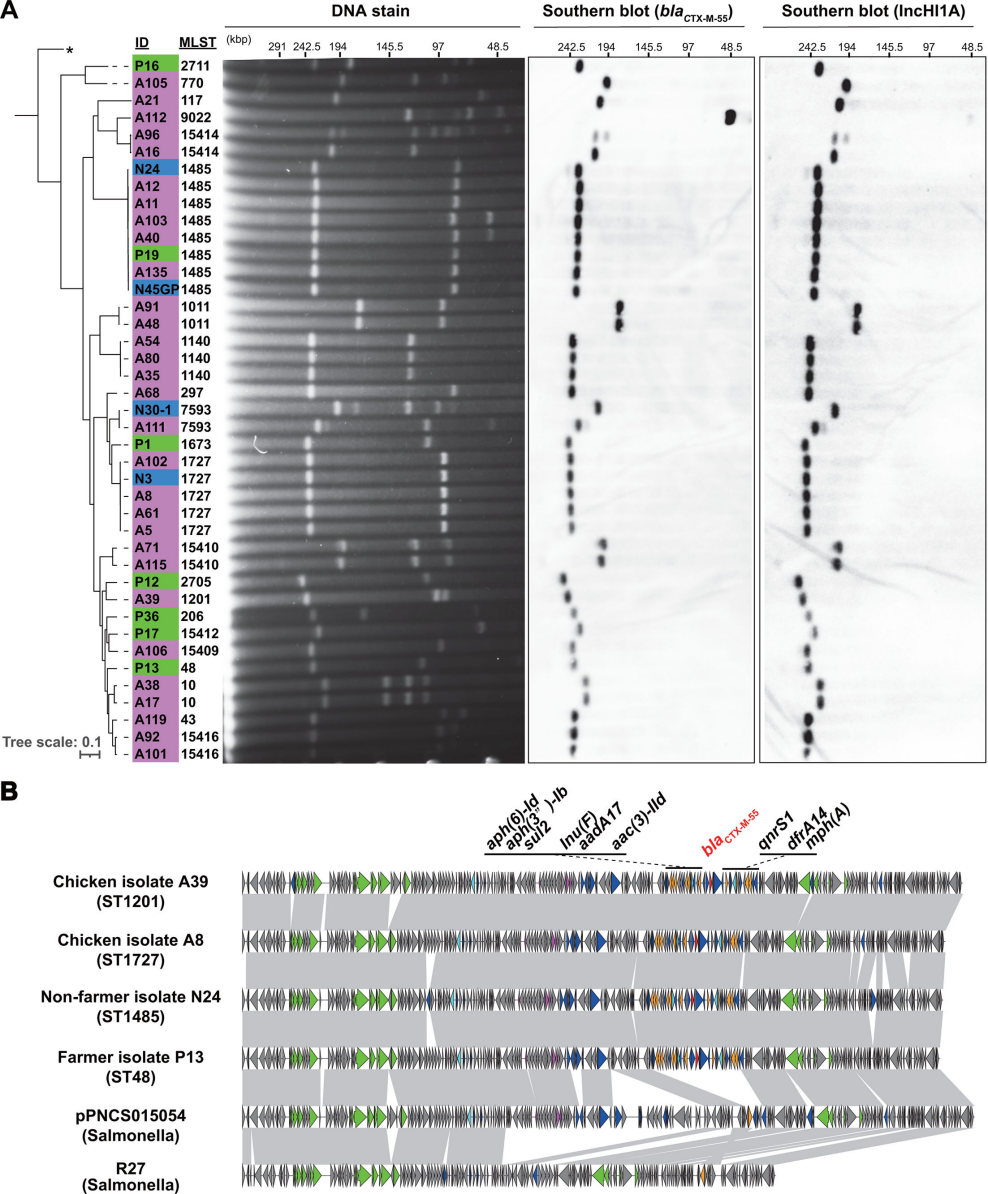
IncHI1-type plasmid harboring *bla*_CTX-M-55_ spread among different STs. (**A**) S1-PFGE followed by Southern blot analysis of the isolates carrying *bla*_CTX-M-55_. Isolate IDs originating from chicken, farmer, and non-farmer were marked magenta, green, and blue, respectively. A phylogenetic tree of the isolates carrying *bla*_CTX-M-55_ was constructed by CSI Phylogeny (https://cge.food.dtu.dk/services/CSIPhylogeny/). Asterisk denotes an *Escherichia fergusonii* strain FDAARGOS_1499 (GCF_020097475.1), which was included as an outgroup. (**B**) Whole plasmid structures of IncHI1 plasmid harboring *bla*_CTX-M-55_ identified in this study (A39, DDBJ/ENA/GenBank accession number LC851332; A8, LC851333; N24, LC851334; P13, LC851335) and structurally related plasmids available from the GenBank database (pPNCS015054, CP037875.1; R27, AF250878.1). Genes are depicted by arrows. The *bla*_CTX-M-55_ gene, red; other antimicrobial resistance genes, orange; integrase gene, cyan; other mobile genetic elements, blue; conjugal transfer genes, green; tellurium resistance genes, magenta; other genes, dark gray. Homologous regions (>99% identity) are shaded in gray. PFGE, pulsed-field gel electrophoresis; ST, sequence type.

As previously described, *bla*_CTX-M-55_ was the most prevalent ESBL gene and was identified in 22 distinct STs (Fig. 1), indicating its dissemination beyond the clonal barrier. S1 nuclease-pulsed field gel electrophoresis (S1-PFGE) and Southern blot analysis demonstrated that the majority of *bla*_CTX-M-55_-positive isolates harbored this gene on a plasmid approximately 200 kb in size (Fig. 6A). Long-read sequencing of representative isolates revealed that the gene was located on a plasmid carrying the IncHI1A, HI1B, and FIA(HI1) replicons. This plasmid also co-harbored resistance genes against aminoglycosides (*aac(3)-IId*, *aph(6)-Id*, *aph(3’’)-Ib*, and *aadA17*), macrolides (*mph(A*)), lincosamide (*Inu(F*)), quinolones (*qnrS1*), sulfonamide/trimethoprim (*sul2* and *dfrA14*), in addition to *bla*_CTX-M-55_. Transconjugants of chicken, farmer, and non-farmer isolates carrying this plasmid showed increased minimum inhibitory concentrations for gentamicin, levofloxacin, and sulfamethoxazole/trimethoprim, in addition to cephalosporins, compared with those of the parental *E. coli* strain (Table S1), showing that this plasmid confers multidrug resistance. The genetic structures of plasmids from the four isolates of different STs and origins were identical, with the exception of several insertions and deletions of insertion sequences and genes encoding hypothetical proteins (Fig. 6B). Genomic analysis revealed that all *bla*_CTX-M-55_-positive isolates, except A112 (*n* = 40), harbored IncFIA(HI1), IncHI1B(R27), and IncHI1A replicons. S1-PFGE and Southern blot analyses targeting the IncHI1A replicon showed that *bla*_CTX-M-55_-positive plasmids were also positive for the IncHI1A replicon in all but one isolate (A112), indicating that this type of plasmid is a major vector for the dissemination of antimicrobial resistance in our isolates. The plasmid contained the complete backbone of the prototypical IncHI1-type plasmid and the R27 plasmid of *Salmonella* origin (17). A BLAST search of the NCBI database using the plasmid from isolate A8 as a query revealed that the pPNCS015054 plasmid from *Salmonella* isolate, PNCS015054, which was obtained during an investigation of human food poisoning cases (18), was the most similar, with 84% coverage and 99.9% identity. This suggests that Lombok plasmids carrying *bla*_CTX-M-55_ originated from plasmids of *Salmonella* origin.

## DISCUSSION

Indonesia is an upper middle-income country in Asia where the dissemination of AMR represents a substantial public health concern. A study estimated that 66.4% and 58.1% of *E. coli* isolates from hospitals and communities, respectively, exhibited resistance to third-generation cephalosporins (19). To date, several molecular epidemiological studies on ESBL have been conducted in Indonesia. A hospital surveillance conducted in Surabaya, Indonesia in 2010 revealed that 94.5% of ESBL-producing *E. coli* isolates were positive for *bla*_CTX-M-15_ (20). Another molecular epidemiological study conducted in Surabaya on healthy medical students demonstrated that *bla*_CTX-M-55_ (50.0%) and *bla*_CTX-M-15_ (34.1%) were the most and second most prevalent ESBL-Ec types, respectively (21). A surveillance study of pregnant women in Surabaya also identified the presence of these two ESBL genes among ESBL-Ec, with *bla*_CTX-M-15_ being the most prevalent (30.6%) and *bla*_CTX-M-55_ being the second most prevalent (16.5%) (22). However, little information is available regarding the prevalence of ESBL genes in Indonesian poultry isolates. A recent molecular epidemiological study on ESBL-producing *E. coli* in cloacal isolates from broiler chickens in the East Java Province revealed that 29.6% were phenotypically ESBL producers, and 94.1% were positive for the *bla*_CTX-M_-type gene (23). Another study reported that 4 of 38 multidrug-resistant *E. coli* isolates from broiler chickens in West Java Province were positive for *bla*_CTX-M_ and *bla*_TEM_ (24). Nevertheless, information on the genetic or genomic characterization of these ESBL-Ec isolates is limited, and no comparative genomic studies of ESBL-Ec in different sectors have been conducted in Indonesia.

To the best of our knowledge, this study is the first to demonstrate that *bla*_CTX-M-55_ and *bla*_CTX-M-15_ are prevalent in human and chicken commensal *E. coli* isolates in Lombok. The distribution pattern of *bla*_CTX-M-55_ is noteworthy. It was identified in 20% (*n* = 41/200) of the total isolates, found in 22 different STs, and predominantly found in chicken isolates (*n* = 30/41). Our findings demonstrated that an approximately 200 kb IncHI1 plasmid was the primary vector for the *bla*_CTX-M-55_ gene. The IncHI1 plasmid is a broad host range conjugative plasmid with an optimal temperature for conjugal transfer at 22°C–30°C. This suggests that its potential dissemination among bacterial species in water and soil environments is more likely than inside animal bodies (25). Moreover, the presence of a gene encoding a global transcriptional regulator, the H-NS-like protein, on this plasmid enables it to enter new bacterial hosts with minimal host fitness cost (26). These characteristics may underlie our observation that the IncHI1 plasmid harboring *bla*_CTX-M-55_ is prevalent and found in a variety of *E. coli* genotypes in both humans and chickens. Notably, the plasmid identified in this study co-harbored a number of antimicrobial resistance genes, including those conferring resistance to aminoglycosides and quinolones. The spread of this plasmid may reduce the therapeutic options for infections caused by pathogens. Therefore, vigilance is necessary for its further spread.

The *bla*_CTX-M-15_ gene was the second most prevalent ESBL gene in this study, accounting for 64.7% (*n* = 11/17) of the human isolates. It was the most predominant gene in human isolates carrying ESBL/AmpC (*n* = 11/29), along with *bla*_CTX-M-55_. Seven of the 17 *bla*_CTX-M-15_-carrying isolates belonged to ST2690; thus, this strain was a major vehicle for the spread among chickens and humans. In addition, this gene was found in seven other STs, suggesting that plasmids are involved in the spread of the gene, as has been found in many other studies (4). However, this was not addressed in this study.

A phylogenetic analysis of the *E. coli* isolates from Lombok revealed the presence of a diverse range of *E. coli* strains, each with a distinct genetic profile, when considered in a global context. Globally successful ESBL-producing strains, such as ST131 and ST1193 (3), have not been identified among isolates. The most prevalent STs among those carrying the ESBL genes in this study were ST48, ST2690, ST1485, and ST1727. ST48 isolates in Lombok were isolated from chicken and human, and three distinct β-lactamases, *bla*_CTX-M-1_, *bla*_CTX-M-55_, or *bla*_CMY-2_, were found in these isolates. The *bla*_CTX-M-1_ gene is associated with a human-specific cluster, whereas the other two genes are found separately in the phylogenetic tree (Fig. 2). Phylogenetic studies have indicated that ST48 has been isolated from a variety of sources, including human, animal, and environmental samples, and has been reported to be associated with various ESBL and carbapenemase genes (27–30), suggesting its potential to adapt to diverse environments and acquire exogenous genes. ST1485 is a member of the clinically important phylogroup F, has been identified as a globally disseminated, high-risk, virulent, and multidrug-resistant clone, and has been isolated from a range of sources, including human, animal, and food samples (31). In contrast, ST2690 and ST1727 have been previously reported in poultry (32–34). It can be reasonably inferred that these strains have the potential to adapt to humans and chickens and are likely indigenous to Lombok. Lombok ST1485 isolates carrying *bla*_CTX-M-55_, ST1727 carrying *bla*_CTX-M-55_, and ST2690 carrying *bla*_CTX-M-15_ were present in samples from humans and chickens, and these clones were phylogenetically indistinguishable from one another. It is reasonable to suspect that the close interaction between poultry farmers and their livestock in the context of indigenous chicken farming may contribute to the bidirectional transmission of these clones. Nevertheless, the detection of identical clones in non-farmers for the above three STs suggests that transmission pathways may extend beyond direct contact with poultry. This has prompted questions regarding the broader mechanisms of resistance dissemination, including environmental contamination or indirect transmission routes. Furthermore, this finding underscores the necessity for a comprehensive approach to surveillance and intervention that considers the intricate interrelationships between humans, animals, and their shared environments.

Notably, carbapenemase and colistin-resistant *mcr* genes were not detected in the course of our screening process, and all ESBL-Ec isolates were susceptible to meropenem and colistin in our antimicrobial susceptibility testing (Fig. S2), thereby sparing treatment options for infections caused by ESBL-Ec found in this study. However, it is conceivable that these AMR pathogens were not identified due to the absence of a selective medium for bacterial isolation. Similarly, ESBL-Ec strains that were not dominant in humans and chickens may not have been detected in our screening process (35). More targeted approaches are required to gain a comprehensive understanding of the AMR status in healthy Lombok residents and chickens. Notwithstanding these limitations, our findings indicated the presence of specific ESBL-Ec strains and a plasmid carrying an ESBL-encoding gene in human and chicken populations in this region. The precise mode of transmission of ESBL-Ec strains, the route of spread of plasmids encoding ESBL genes, and their impact on regional public health remain unclear. Further studies employing a One Health approach are required to elucidate the dissemination of AMR pathogens on Lombok Island and develop strategies to mitigate their further spread.

## MATERIALS AND METHODS

### Bacterial isolation

Healthy human participants were randomly selected from urban (non-farmer) and rural (farmer) areas of Lombok in August 2021. Signed informed consent for sample collection was obtained from all participants, and sample collection was performed by the participants themselves. Rectal swabs from farmers and non-farmer volunteers (*n* = 107) and cloacal swabs from chickens (*n* = 155) were grown on eosin methylene blue (EMB) and MacConkey (MAC) agar. Metallic green and colorless colonies on EMB and MAC, respectively, were subjected to indole, methyl red, Voges–Proskauer, and citrate (IMViC) tests to identify *E. coli*. Isolates identified as *E. coli* were transferred to the National Institute of Infectious Diseases (NIID), Tokyo, Japan.

### Whole-genome sequencing and bioinformatic analysis

A total of 200 *E. coli* isolates were successfully cultured after transportation to NIID and subsequently subjected to whole-genome sequencing using the Illumina MiSeq, MiniSeq, or HiSeq X Five platforms, and some were additionally analyzed by long-read sequencing using the GridION system (Oxford Nanopore Technologies) to complete plasmid sequences. Library preparation was performed as previously described (36). Briefly, an Enzymatics 5 × WGS fragmentation mix and WGS ligase reagents (Qiagen) were used to construct an Illumina library. Nanopore sequencing libraries were prepared using the Rapid Barcoding Kit, and sequencing was carried out using the R9.4.1 flow cell. Base calling was performed using Guppy (v4.0.11–v5.0.12), and hybrid assembly was conducted using Unicycler (v0.4.8) (37).

Detection of antimicrobial resistance gene and plasmid typing was performed by abricate (https://github.com/tseemann/abricate) using ResFinder (38) and PlasmidFinder (39) databases, respectively. MLST was done by mlst 2.23.0 (https://github.com/tseemann/mlst). In phylogenetic analysis, we first created a core-genome SNP-based maximum likelihood (ML) tree for all isolates from Lombok Island using RAxML-NG v1.1 with the GTR+Γ model (40). Next, we constructed four phylogenetic trees for ST48, ST1485, ST1727, and ST2690 using publicly available sequences. Isolates belonging to ST48, ST1485, ST1727, and ST2690 were searched using EnteroBase (https://enterobase.warwick.ac.uk/). Raw sequencing reads of the hit isolates were downloaded from the GenBank database and analyzed. We constructed an ST48 tree using a neighbor-joining method with ClustalW version 2.0 (41). To construct the other three trees (ST1485, ST1727, and ST2690), we first mapped trimmed reads to each reference sequence (ST1485, GCP042896.1; ST1727, chicken isolate A8 [completed in this study]; and ST2690, AP025220.2) using Snippy v4.6.0 with default parameters (https://github.com/tseemann/snippy). After concatenating the Snippy outputs and creating aligned FASTA files, we reconstructed ML trees using Gubbins v3.1.3, with 100 bootstrap replicates (42). All trees were midpoint-rooted and visualized using iTOL (43). To calculate the average pairwise SNP distance within each ST, contigs were first annotated using Prokka v1.14.6 (44). Core-genome identification was then performed using Panaroo v1.3.4 (45) with default parameters. Finally, an SNP distance matrix was generated using snp-dists v0.8.2 (https://github.com/tseemann/snp-dists).

### Plasmid analysis and antimicrobial susceptibility testing

Isolates positive for the *bla*_CTX-M-55_ gene were analyzed using S1-PFGE followed by Southern blot analysis, as described previously (46). Transconjugants of isolates carrying the plasmid with *bla*_CTX-M-55_ were obtained using *E. coli* ML4909 (47) as the recipient. A mating assay was conducted on nitrocellulose membranes on a Mueller–Hinton agar plate by incubating at 30°C for 6 h. Transconjugants were selected on a brain heart infusion agar plate supplemented with 4 µg/mL cefotaxime and 100 µg/mL rifampicin.

The MICs of isolates positive for ESBL/AmpC genes and transconjugants were determined using the MicroScan WalkAway and NEG MIC 3 J panels (Beckman Coulter Inc.). The results were interpreted according to the Clinical and Laboratory Standards Institute guidelines (48).

## Data Availability

The raw sequencing reads obtained in this study were submitted to the DDBJ/ENA/GenBank database under the BioProject number PRJDB18129. Accession numbers for each isolate obtained in this study are listed in Table S2.

## SUPPLEMENTAL MATERIAL

Supplemental material aem.02364-24-s0001.docx Figures S1 to S4; Table S1.

Table S2 aem.02364-24-s0002.xlsx Details of the isolates analyzed in this study.

ASM does not own the copyrights to Supplemental Material that may be linked to, or accessed through, an article. The authors have granted ASM a non-exclusive, world-wide license to publish the Supplemental Material files. Please contact the corresponding author directly for reuse.
