# Supplementary material for: Genomic characterization of extended-spectrum β-lactamase-producing Escherichia coli spread among chickens and healthy residents in Lombok, Indonesia
Source: Appl Environ Microbiol. 2025 Apr 11;91(5):e02364-24. doi: 10.1128/aem.02364-24 (PMC12093981; doi:10.1128/aem.02364-24)
Supplement: Supplemental material — Figures S1 to S4; Table S1. [file aem.02364-24-s0001.docx]

**Fig S1**

**Fig S1** Venn diagram showing the MLST sequence types found in and shared among chicken, farmer and non-farmer isolates in this study.

**Fig S2**

**Fig S2** The susceptibility rate of chicken isolates was higher than that of human isolates for gentamicin, tobramycin, levofloxacin and minocycline. (**A**) The susceptibility rate of chicken and farmer/non-farmer isolates that carry the ESBL/AmpC gene for β-lactam antimicrobials. (**B**) The susceptibility rate of chicken and farmer/non-farmer isolates that carry the ESBL/AmpC genes for other class of antimicrobials. (**C**) The susceptibility rate of the isolates with *bla*_CTX-M-55_ or other ESBL/AmpC gene for indicated antimicrobial (**D**) Carriage rate of antimicrobial-resistant determinants in chicken and farmer/non-farmer isolates that carry the ESBL/AmpC gene.

**Fig S3**

**Fig S3** Isolation source of the *E. coli* ST48, 1485, 1727, and 2690 strains that were publicly available from the NCBI database and were included in the phylogenetic analysis in this study.

| **Fig S4**  ****  **Fig S4** Single nucleotide polymorphism (SNP) differences between chicken, human, and between chicken and human ST1485 (A), ST1727 (B), and ST2690 (C) isolates obtained in this study.  **Table S1** Minimum inhibitory concentrations of isolates carrying IncHIA-type plasmid (A8, P13, N24) with *bla*_CTX-M-55_ and their transconjugants (A8TC, P13TC, N24TC). ML4909 is a recipient strain used in this experiment. | | | | | | | | | | | | | | |  |
| --- | --- | --- | --- | --- | --- | --- | --- | --- | --- | --- | --- | --- | --- | --- | --- |
|  |  | |  | | |  | |  | |  | |  | |  |  |
| **Antimicrobial agents** | **MICs (mg/L)** | |  | | |  | |  | |  | |  | |  |  |
|  | | **A8** | | **P13** | **N24** | | **A8**  **TC** | | **P13**  **TC** | | **N24**  **TC** | | **ML4909** | | |
| **Cefpodoxime** | | >4 | | >4 | >4 | | >4 | | >4 | | >4 | | ≦2 | | |
| **Cefotaxime** | | >32 | | >32 | >32 | | >32 | | >32 | | >32 | | ≦1 | | |
| **Ceftriaxone** | | >32 | | >32 | >32 | | 32 | | 32 | | >32 | | 2 | | |
| **Ceftazidime** | | 8 | | 16 | >16 | | ≦1 | | 4 | | 4 | | ≦1 | | |
| **Aztreonam** | | >16 | | >16 | >16 | | <4 | | 8 | | >16 | | ≦4 | | |
| **Cefepime** | | >16 | | >16 | >16 | | 4 | | 16 | | 8 | | ≦2 | | |
| **Gentamicin** | | >8 | | >8 | >8 | | >8 | | >8 | | >8 | | ≦4 | | |
| **Levofloxacin** | | 0.5 | | 0.5 | >4 | | 0.5 | | 0.5 | | 0.5 | | ≦0.12 | | |
| **Minocycline** | | ≦4 | | ≦4 | 8 | | ≦4 | | ≦4 | | ≦4 | | ≦4 | | |
| **Sulfamethoxazole/ Trimethoprim** | | > 2/38 | | > 2/38 | > 2/38 | | > 2/38 | | > 2/38 | | > 2/38 | | ≦2/38 | | |
